# Facilitators and barriers to using a DeskCycle as a sedentary behavior intervention in the work environment

**DOI:** 10.1371/journal.pone.0299537

**Published:** 2024-03-14

**Authors:** Lorriane A. Odhiambo, Alexander J. Marion, Alison E. Harmatz, Joy A. Yala, Thomas R. Callihan, Kristina Bundy, Melissa D. Zullo

**Affiliations:** 1 Department of Biostatistics, Data Science and Epidemiology, Augusta University, Augusta, GA, United States of America; 2 Medical College of Georgia, Augusta University, Augusta, GA, United States of America; 3 College of Public Health, Kent State University, Kent, OH, United States of America; University of Costa Rica, COSTA RICA

## Abstract

**Background:**

Sedentary behavior is a public health threat with extensive health burden on society. High levels of sedentary behavior have been associated with cardiovascular diseases, diabetes, obesity, and cancer. Individuals working in desk-related occupations are more likely to be sedentary for most of the day. Health researchers have responded by implementing and promoting interventions and wellness programs in work environments to reduce this behavior. This study examined the feasibility and experience of using the DeskCycle to reduce sedentary behavior among female workers in an academic office environment.

**Methods:**

This was an intervention study where participants used the DeskCycle in two consecutive eight-week phases and uploaded DeskCycle use data daily. A questionnaire was administered after week 2 and week 8 (pre-post) of DeskCycle use in each phase to assess dimensions of feasibility, including an open-ended question for user experience.

**Results:**

The participants (N = 78) had an average age of 44.4 (±11.3) years and were primarily non-Hispanic White (88.5%). DeskCycle daily use varied from Phase I: 84% to 64.9% (weeks 1–7), and 49.4% in week 8, to Phase II: 73.5% to 52.2% (week 1–7), and 40.2% in week 8. In Phase I, 96.6% (week 2) and 87% (week 8) agreed that the DeskCycle decreased sedentary behavior, and in Phase II, 74.3% (week 2) and 76.9% (week 8) agreed. The analysis of open-ended responses found challenges with the desk set up, cycling interfering with typing, and thinking critically, as barriers to DeskCycle use, while enjoying cycling and cycling improving mood were reported as facilitators.

**Conclusions:**

Using a DeskCycle in an academic office environment to reduce sedentary behavior is feasible in female office workers. Consideration should be given to the type of tasks performed while cycling.

## Introduction

Sedentary behavior (SB) is a significant public health issue with increasing prevalence as lifestyles modernize. SB is independently associated with multiple unhealthy behaviors, obesity, type II diabetes, cardiovascular disease, cancers, and all-cause mortality [1–6]. Rates of SB are increasing with roughly 25–35% of Americans in 2009 being completely sedentary [7]. Trends remained relatively high and stable through 2016 [8]. This increase is primarily driven by an upsurge in sedentary jobs, as researchers with the American Heart Association report an 83% increase in such jobs since the 1950’s [5]. Typically, desk employees are sedentary 66–82% of their day, meaning much of the workday is spent at the desk, without taking adequate breaks to stand up or walk around [1, 9]. Research has found that rates of increased SB hold even when controlling for level of physical activity. This means an individual may exercise regularly yet engage in high levels of SB, increasing their risk for negative health outcomes [10]. Individuals engaging in 8 hours or more of SB each day are more likely to need 300 instead of the recommended 150 minutes of moderate exercise each week to avoid adverse outcomes [11, 12]. SB is defined as any waking behavior characterized by an energy expenditure ≤1.5 metabolic equivalents (METs), while sitting, reclining, or lying down [13]. For reference, 1 MET is defined as the energy expended when an individual is resting or sitting still. It is calculated as a ratio; for example, 6 METs in moderate exercise indicates an individual is expending six times the energy used when resting or sitting.

Appropriately, a growing body of literature aims to develop and implement interventions to reduce SB, particularly in the workplace. Some studies have examined modifications to the desk setup or workstation, with specific interventions including the standing desk, treadmill desk, and under desk bikes. There are fewer studies on under desk bikes, and more evidence for benefit in the standing desk and treadmill desk reducing SB [14–16]. However, the standing desk fails to boost METs as the treadmill desk does, and both interventions can be expensive [17]. Some evidence suggests the treadmill desk interferes with work performance, potentially interfering with typing capabilities at higher speeds [18]. Previous under desk bike studies assessed user experiences at one timepoint [19, 20], post-intervention, and had modest sample sizes. However, findings showed participant engagement and reduction of sedentary time in the workplace [21], positive experiences linked to attention, work performance and motivation [19], and improvement in cardiometabolic markers such as total cholesterol [22]. These findings are important and comparing user experiences at two timepoints could further our understanding of the long-term utility of under desk bikes in the office environment. This study examined the feasibility and experiences of female employees using an under desk bike, the DeskCycle, to reduce SB in an academic office environment. Despite the identified barriers, our findings showed that most participants still realized important benefits that give evidence for the feasibility of using the DeskCycle to reduce SB in an office work environment.

## Methods

### Study design

This was a pre-post study within a larger randomized controlled trial (RCT) with a crossover design [23]. As part of the RCT, participants were randomly assigned to use the DeskCycle in one of two sequential eight-week phases separated by a 7-week washout period. The DeskCycle is an under desk bike with eight resistance levels, and a five-function 1000-minute display that tracks speed, time, distance, calories, and scan. At the start of each Phase, participants received a DeskCycle, user’s manual, setup toolkit, and study-developed handout containing instructions on resistance level, suggested usage patterns, distance reading, time tracking, and troubleshooting. Each participant was assigned a trained research assistant (RA) to serve as a point of contact to address any problems during the study period (**explained further in RCT publication [23]**). Quantitative and qualitative data were collected using a feasibility questionnaire from October 2017 to March 2018. Due to the nature of the intervention, blinding was not conducted, hence the researchers had access to participant identifying information during project implementation. The Kent State University Institutional Review Board (IRB# 17–226) approved this study and all participants provided written consent.

### Sample and eligibility

The study was conducted over six months among employees at a university in the Midwest US. To be eligible, participants had to be full-time employees at the University, self-report being sedentary >75% of their workday and work a desk/computer-dependent job. A significant proportion of the administrative employees were women; hence, the sample was limited to this sub-group. Additionally, based on recommendations from the DeskCycle manufacturer, participants had to be less than 5’10” in height and have a workplace desk height of greater than 27”. Individuals with shorter desks or a greater height are more likely to experience discomfort and difficulty using the machine. Potential participants with pre-existing musculoskeletal problems and cardiometabolic diseases were excluded.

### Recruitment

Recruitment was conducted using a screening questionnaire sent by email to 1095 university employees in September of 2017. The email was sent twice over two weeks. It included questions on the eligibility criteria, demographics, participant availability, participation in the Move Challenge, and previous use of a standing desk. The Move Challenge is an annual, campus-wide wellness initiative intended to incentivize employees to be more physically active.

A total of 479 (43.7%) employees responded to the questionnaire. A review of the screening data found 261 eligible participants (54.5%), from which 80 employees were randomly selected for the larger study using simple randomization. The sample (N = 80) was determined by the number of DeskCycles available for the study (40). Trained RAs enrolled the participants and collected baseline data. The first group of participants used the DeskCycle in October and November of 2017 (Phase I), and the second group used the same machines in February and March of 2018 (Phase II).

### Outcome measure

Feasibility and user experience were assessed using a modified questionnaire from a previous study [20] and DeskCycle use data uploaded daily by participants using an online form [23]. The form included two questions ‘Did you use the DeskCycle today?’ followed by ‘If no, what was the main reason for not using the DeskCycle?’ The study questionnaire that primarily assessed user experience included 13 of the original 23 questionnaire items from the previous study [20]. The items were formatted as statements and responses on a five-point Likert scale that ranged from strongly disagree to strongly agree. The questionnaire was intended to assess participants’ attitudes towards the DeskCycle across a variety of dimensions and impact on physical activity level to give a holistic picture of using the machine in the workplace. In statements where ‘agree’ was a positive response, participants were asked whether, ‘the DeskCycle was convenient to use, they were comfortable using it around others, it decreased SB, physical activity outside of work increased during use, it was an alternative for exercise during bad weather for example, during the winter, the real-time monitor increased use, and they would use it if offered by employer.’ In statements where ‘disagree’ was a positive response, participants were asked whether, ‘the DeskCycle was noisy, their work productivity decreased, their work quality decreased, it interfered with work-related tasks, it caused physical discomfort, and physical activity outside of work decreased.’ The final item on the questionnaire was an open-ended question used to solicit responses about participant experience using the DeskCycle ‘Do you have any additional comments about your experience using the DeskCycle?’ Data was collected at four time points (2, 4, 6, and 8 weeks) in both Phase I and Phase II. Only the data from time points 2 and 8 were analyzed to reflect experiences at the beginning and end of DeskCycle use.

### Statistical analysis

This was a pre-post analysis with a qualitative component. Demographic data (**Table 1**) was summarized using frequencies, proportions, means and standard deviations. To estimate compliance with DeskCycle use, all participant daily entries were reviewed for ‘Yes’ responses to the question asking whether they cycled that day. This review was conducted prior to excluding participants that reported ≤2 days/week and ≤4 weeks/phase of DeskCycle use from non-sedentary time calculations [23]. The responses were aggregated for each week (1–8) to calculate the proportion of participant entries that reported DeskCycle use.

**Table 1:** Descriptive characteristics of DeskCycle intervention participants.

Participant characteristics	f (%)
Age (years) (M, SD)	44.4 (±11.3)
Baseline BMI (Kg/m^2^) (M, SD)	30.9 (±8.0)
Baseline Mood Score (M, SD)	19.9 (±9.7)
Total Weekly PA (min.) (M, SD)	137.4 (±78.4)
Weekly PA Frequency (days)	3.7 (±1.7)
Education Level	
High school/Some college	19 (24.4)
Undergraduate degree	25 (32.1)
Graduate degree	34 (38.6)
Race	
Non-Hispanic White	69 (88.5)
Other	9 (11.5)
Move Challenge	
Yes	47 (60.3)
No	31 (39.7)
PA Tracking Device	
Yes	42 (53.8)
No	36 (46.2)
Light to moderate PA	
Yes	68 (87.2)
No	10 (12.8)

Abbreviations: Mean (M); Minutes (Min); Physical Activity (PA)

Standard Deviation (SD)

To provide descriptive statistics for DeskCycle user experience, the five-point Likert scale responses (strongly Disagree-1, disagree-2, neutral-3, agree-4, strongly agree-5) to each statement were collapsed into two main categories, ‘agreed (4 or 5)’ or ‘disagreed (1 or 2)’. The neutral responses (3) were excluded from analysis. The proportion of participants that responded to ‘agree’ or ‘disagree’ were calculated for statements 1–13 from weeks 2 and 8 of each Phase. The Wilcoxon signed rank test for paired non-parametric data was used to assess whether the median difference between week 2 and week 8 responses in each phase were equal to zero. This test was used since our data is dependent and not normally distributed, but rather ordinal in nature. A p-value of <0.05 was considered significant, and analysis was conducted using SAS version 9.4 (SAS Institute Inc., Cary, NC).

#### Analysis of open-ended survey responses

More than half of the participants from each time point contributed to a total of 65 responses to the open-ended question. Specifically, in phase I, 18/29 participants (62%) and 15/23 (65.2%) responses in weeks 2 and 8, and in phase II, 19/35 (54%) and 13/26 (50%) responses in the respective weeks. The responses were analyzed to determine themes. This involved creating a short codebook and using the codes to generate sub-themes and ultimately themes from the data. Sub-themes were generated by one author through identifying the most frequently mentioned ideas or codes within the responses [24, 25]. The themes were then reviewed by a second author for verification.

## Results

Participants were predominantly non-Hispanic White and well educated, with approximately three-quarters of the sample reporting an undergraduate degree or higher (**Table 1**). Participants had an average age of 44.4 (±11.3) years and a body mass index (BMI) of 30.9 (±8.05) Kg/m^2^. They reported engaging in physical activity approximately three times per week, with an average of 137.4 (±78.4) minutes spent on such activities. Of the sample, 60.3% reported participating in the university’s Move Challenge.

DeskCycle compliance, which was a ‘Yes’ response to the question ‘Did you use the DeskCycle today?’ ranged from 84% (week 1) to 64.9% (week 7) in phase I, then decreased to 49.4% in week 8. A similar pattern was noted in phase II, but with lower proportions, 73.5% (week 1) to 52.2% (week 7) and decreased to 40.2% in week 8. The main reasons given by participants for not using the DeskCycle included absence from the office, being busy, having meetings all day, not feeling like using it, and being sick or unwell.

**Table 2** shows the results for agreement with questionnaire statements. Results suggest that the majority of participants felt the DeskCycle is feasible as a workplace intervention to help reduce SB. Participants consistently agreed with positive aspects of DeskCycle use and disagreed with negative aspects. Overall, these findings held both within phases from week 2 to week 8 and between phases when comparing Phase I to Phase II. Notable trends include a decline from 96.6% to 87% agreement in Phase I compared with a modest increase from 74.3% to 76.9% agreement in Phase II about the DeskCycle reducing SB in the workplace. Additionally, when asked whether they would use the DeskCycle if offered by an employer, 86.2% agreed in week 2 of phase I, with a decline to 73.9% by the end of week 8. In Phase II, 88.1% agreed in week 2 which declined to 73.1% in week 8. Participants disagreed that work quality was diminished when using the DeskCycle, Phase I (week 2: 82.8%, week 8: 91.3%), and Phase II (week 2: 71.4%, week 8: 69.2%).

**Table 2:** DeskCycle users experience at the end of weeks two and eight in both phases.

	Phase I		Phase II	
	Week 2 (n = 29)	Week 8 (n = 23)	Week 2 (n = 35)	Week 8 (n = 26)
**Statements**	%			
**Agreed**				
Convenient to use	72.4	73.9	65.7	69.2
Comfortable use around others	65.5	72.7	65.7	76.9
Decreased sedentary behavior	96.6	87.0	74.3	76.9
PA outside work increased	27.6	26.1	8.6	30.8
Alternative during bad weather	93.1	82.6	82.9	73.1
RT monitor increased use	72.4	78.3	60.0	50.0
Offered by employer	86.2	73.9	88.6	73.1
**Disagreed**				
Noisy	96.5	91.3	88.6	73.1
Work productivity decreased	75.9	60.9	60.0	69.2
Work quality decreased	82.8	91.3	71.4	69.2
Interfered work-related tasks	62.1	60.9	65.7	61.5
PD (back, muscle, or joint pain)	82.8	82.6	85.7	73.1
PA outside work decreased	75.9	69.6	68.6	65.4

Abbreviations: Physical Activity (PA); Physical Discomfort (PD); Real-Time (RT); Number of participants that completed the questionnaire (n)

The Wilcoxon signed rank test revealed significant differences in both phases for the statement on whether participants would ‘use the DeskCycle if offered by their employer’ (**Table 3**). The negative differences suggest a significant difference in post-test ranks, week 2 compared to week 8, Phase I, (W = -10.5, p-value = .0313), and Phase II, (W = -24, p-value = .0313). A significant difference was also found in phase II for the statement on whether ‘physical activity increased outside of work’, (W = 0.43, p-value = .0437). The positive difference indicates that the post-test ranks in week 8 were significantly higher than week 2.

**Table 3:** Wilcoxon signed-rank test comparing responses on DeskCycle use from weeks two and eight.

	Phase I			Phase II		
Statements	Mean Difference (SD)	WRS TS	WRS p-value	Mean Difference (SD)	WRS TS	WRS p-value
**Agree**						
Convenient to use	-0.11 (0.88)	-3.5	.7891	-0.13 (0.76)	-7.5	.5898
Comfortable use around others	-0.16 (1.17)	-6	.6133	0.17 (0.83)	5.5	.4688
Decreased sedentary behavior	-0.21 (0.85)	-7	.4375	0.09 (0.95)	2.5	.8281
PA outside work increased	-0.11(0.74)	-3.5	.7656	0.43 (0.9)	29	.0437*
Alternative during bad weather	-0.32 (0.75)	-8	.1563	-0.39 (1.31)	-11.5	.2148
RT monitor increased use	-0.32 (1.12)	-11	.3223	0.04 (0.93)	2.5	.875
Offered by employer	-0.53 (0.9)	-10.5	.0313*	-0.52 (0.99)	-24	.0313*
**Disagree**						
Noisy	0.21 (0.42)	5	.125	0.26 (0.62)	10.5	.1094
Work productivity decreased	0.37 (0.76)	17.5	.0918	-0.26 (0.96)	-13	.3047
Work quality decreased	0.05 (0.71)	1.5	1	-0.04 (0.82)	-4	1
Interfered work-related tasks	0.32 (1.42)	12	.4285	-0.13 (0.81)	-9	.6133
PD (back, muscle, or joint pain)	0.05 (1.13)	0.5	1	0.13 (0.97)	5	.6836
PA outside work decreased	-0.05 (1.35)	1.5	.8711	0.04 (0.82)	3	1

Abbreviations: Physical Activity (PA); Real-Time (RT); Physical Discomfort (PD); Wilcoxon Rank Signed Test Statistic (WRS TS); *p-value* <0.05

The analysis of open-ended responses yielded two broad themes, barriers to DeskCycle use and facilitators of DeskCycle use. These broad themes were grounded in specific categories of challenges mentioned in the responses such as difficulty using the DeskCycle, impact on work or work quality, and physical activity level. On the other hand, the facilitators were generated from sub-themes including enjoying using the DeskCycle, its positive impact, and a desire to continue use given the opportunity. Sub-themes for barriers included issues with comfort, performing work-related tasks, forgetting to use, and setting up the unit, while facilitators were linked to enjoying cycling, improving activity, and increasing mood. **Table 4** lists themes, sub-themes, and one selected direct quote for each sub-theme.

**Table 4:** Select direct quotes on barriers and facilitators to DeskCycle use.

Quotes	
Sub-themes	Theme: barriers to DeskCycle use
**Difficulty using the DeskCycle**	*"Yes*, *I would love a desk cycle while at work*. *The bad thing as that I would need other equipment to use the desk cycle*. *My desk is too short*, *and knees hit the desk when I tried using it the first time*. *Plus*, *my chair kept rolling back with each push on the pedals*. *The band that was given to hold you in place did not work well and it was hard to get up from my position*, *when need be and back into position*, *when need be*. *So*, *then I was given a standard chair*. *It totally helped with being able to use the cycle SO MUCH BETTER*, *but the chair was too short*, *and I found myself aching in my shoulders from trying to type with a short chair*, *ultimately making my desk to high*. *At this point in time*, *I am not using the cycle at all because it takes too long to get situated and it takes away from work*. *If I could have come up with a way to have a chair that didn’t move that wasn’t low to the ground*, *I think this would have been a completely different experience*. *It’s such a good idea and people who sit long hours should definitely be using it*.*"*
**Negative impact on work**	*“I have a love/hate relationship with the desk cycle*. *It’s an awesome tool and I probably should have taken better advantage of it*. *However*, *it became one more thing on my to-do list*. *Also*, *the majority of my day I hold appointments with students*, *and it is awkward to use the desk cycle while talking to people*. *And it is hard to write or type while using it*. *If I was just talking on the phone or reading something that was when it was easiest to use*.*”*
**Negative impact on physical activity**	*“It became challenging to use the desk cycle as the semester went on for a few different reasons*. *1) I run a lot outside of work and do yoga as cross training*. *Around February is when I started upping my mileage and adding in longer runs and speed workouts*. *Days when I was planning to do a workout or preparing for a longer run*, *I did not want to tire my legs out by using the desk cycle during work*. *2) During the mid-point of the semester is when my office is the busiest due to required advising and other large programs that take place*. *I found myself having less time to use the desk cycle as most of my day was spent with students in my office or I was on the go a lot more planning and preparing for events*. *Overall*, *I really enjoyed using the desk cycle*, *especially as it provided me with great cross training and didn’t require much attention while I worked at my desk on more administrative tasks*.*”*
	**Theme: facilitators of DeskCycle use**
**Enjoy the DeskCycle**	*“I like it very much*. *It keeps your mind on keeping yourself moving even though you’re sitting at a desk all day*. *Thank you for the opportunity to participate*. *I am seriously thinking about getting it for home and using it during TV watching*.*”*
**Positive impact of the DeskCycle**	*“I would love to KEEP this desk cycle*. *I do little or no physical activity at work*. *It’s not a healthy environment for me at all*. *Last evening*, *I felt good about myself because I had such a long day at work*, *got projects finished*, *AND managed to pedal more than 1*.*5 hours on my desk cycle*. *Thank you*!*”*
**Desire for use**	*"Loved the DeskCycle*. *I’ve even looked it up online to see how much it cost*. *I would like to keep going with DeskCycle*. *It took a while to get the correct seating adjustment and tension*. *I found I liked it better on a lower tension setting to keep myself going*. *I didn’t need to concentrate as much to use it*. *Hope this is something that KSU will consider offering at a discount rate to employees*.*"*

## Discussion

This study suggests the DeskCycle is a feasible intervention to reduce workplace SB among women in predominantly sedentary jobs. Participants reported using the DeskCycle in >50% of daily entries for the first seven weeks of each phase. They also consistently agreed with positive statements and disagreed with negative statements about the DeskCycle. Given our pre-post assessment in two phases, important trends were found and are worth exploring, most notably differences from week 2 to week 8 within phases of the study and differences between Phase I and Phase II. For example, 96.6% and 87% of participants agreed that the DeskCycle reduced SB in the workplace in Phase I. However, 74.3% and 76.9% of participants in Phase II agreed with the same statement. Additionally, in week 2 of both phases, 86.2% (Phase I) and 88.6% (Phase II) of participants agreed they would use the DeskCycle if it were offered by their employer. This dropped to 73.9% and 73.1% respectively by week 8. Finally, in Phase I, 82.8% and 91.3% of participants disagreed that their work quality decreased when using the DeskCycle. This was lower in Phase II, as 71.4% and 69.2% of participants disagreed. While both phases overall agreed the DeskCycle was feasible and usable in the office setting, Phase I participants seemed to find the DeskCycle to be more feasible, particularly in the areas of decreasing workplace SB, desire to use if offered by their employer, and belief that their work quality was not impacted by DeskCycle use. One possible explanation for the declines could be that by Phase II, DeskCycle users may have grown accustomed to their peers in Phase I using the cycle and were not as motivated considering participants were located in different buildings across campus, but some were in the same departments. This decline in interest aligned with the reduction in DeskCycle use after the first four weeks in each phase [23]. A mixed-methods study by Torbeyn et al. also reported that participants overwhelmingly agreed to the positive experiences with the under desk bike, despite the reduction in time spent cycling over the study period [19]. This was also the case in an earlier feasibility study [20]. These studies attributed the decline in cycling to the novelty of the intervention wearing off over time [19]. Despite these findings, most participants agreed that the DeskCycle is a feasible intervention for reducing workplace SB with potential adjustments to the user’s experience.

One of the frequently mentioned problems or difficulties with the DeskCycle was modifying the desk set up to allow participants to cycle and work simultaneously. The DeskCycle has recommendations for the ideal desk set up; however, this did not seem to work for all participants. Perhaps the manufacturer guide could include set-up recommendations for people of different heights and weights. Specifically, despite maximum height recommendations for users and minimum desk height requirements, some participants reported still bumping their knees against the desk when cycling. This could be addressed with higher desk height recommendations for people with different body types. Another frequent barrier was ‘forgetting to use the DeskCycle’. This is of interest as an additional area of the literature focuses on behavioral interventions such as reinforcement schedules and reminders to be active to reduce SB [21]. Torbeyn and colleagues provided instructions on weekly cycling to which participants were compliant about 50% of the time [19]. Our study, though lacking a behavioral component, also found compliance to daily DeskCycle use to be relatively above 50% for at least seven weeks. Finally, some participants reported a negative impact of the DeskCycle on critical thinking and typing capability. Although some studies suggest this is broadly not the case, altering participants’ expectations of what work they do while cycling could be useful [15, 18]. In a mixed-methods study, 58% of participants reported no effect, 32% positive, and 11% negative effects on work performance such as difficulty using the computer mouse and making phone calls while cycling [19]. Given this finding, participants could be encouraged to keep practicing cognitive activities while cycling to improve or to do other types of work while using the cycle.

Interestingly, one-third of DeskCycle users in both phases reported that their physical activity level outside of work increased from DeskCycle use, consistent with participants reporting positive influence on health and lifestyle [19]. While this was an unintended effect, it is promising to see physical activity outside of work potentially increase from good workplace habits. This was supported in the open-ended responses as some participants mentioned the DeskCycle being associated with increased physical activity beyond what participants engaged in prior to the study. This could be investigated further in future studies, as increased physical activity outside of the workplace carries significant health benefits.

It is well established that SB is increasing across a variety of employment opportunities, especially in desk and computer-bound jobs [1, 2]. Further, SB is associated with a number of negative health outcomes and behaviors independent of a lack of exercise [5, 6]. Current literature has focused on workplace interventions to reduce SB and support a positive impact on chronic conditions, metabolic diseases, and other health outcomes [15–18, 20]. Reducing SB at work can positively influence healthy behavior outside workhours [6] and is a protective factor for all-cause mortality [26]. Appropriately, interventions to reduce workplace SB are a growing topic of research in primary and secondary prevention. Standing and treadmill desks have been shown to reduce SB though each have documented concerns including price and potential interference with work 15, 18]. The DeskCycle and similar under desk bikes are convenient workplace interventions with limited studies showing acceptability and efficacy in reducing workplace SB [20, 21, 27, 28]. Specifically, previous studies demonstrated the potential of the DeskCycle to engage workers in light to moderate physical activity throughout the day while accomplishing work-related tasks [27, 28].

This is one of the few studies that has examined the feasibility of using an under desk bike among US women working in administrative desk jobs [20, 27]. This study builds on previous data in the literature by increasing sample size and time of use of the cycle [20, 21, 27, 28]. Participants in this study used the DeskCycle at their designated workstations in office buildings across campus, allowing for a real user experience with the DeskCycle while completing usual tasks outside a laboratory environment. This study provided further evidence for the feasibility of using the DeskCycle to reduce SB during work hours in an office setting by assessing experiences at the beginning and end of the intervention. This was informative as we found a decline in cycling after the first four weeks of using the DeskCycle [23], as did other previous studies [19, 20]. The COVID-19 pandemic highlighted the importance of this study and others like it as SB rates increased with more and more people staying home to work rather than going to an office. Zhu et al. found increased rates of weight gain following “Stay-at-Home” mandates from the Chinese government [29]. The two major factors driving weight gain were increased food consumption and decreased physical activity [29]. Additionally, studies suggested that physical activity was essential in combatting the physical and mental tolls of the COVID-19 quarantine [30], highlighting the need for additional studies that investigate DeskCycle efficacy and effectiveness in reducing SB for those working from home or at the office. The DeskCycle is a portable equipment, easily adoptable into an office or the home working environments, without noise or as much cost compared to the treadmill or standing desk.

### Strengths and limitations

This study has several strengths and limitations. One of the major strengths is the sample size, which is larger than similar studies [20, 27]. Specifically, Carr, Walaska, and Marcus used a similar approach with 18 participants from different workplaces [20]. Secondly, the cross-over study design of the larger main study allowed more participants to use the DeskCycle, thus providing more perspectives on their experiences on an intervention with potential benefits [16–18, 20, 21, 27, 28]. Our study included a qualitative aspect that enhanced an understanding of barriers and facilitators linked to the quantitative results. This also allowed for the identification of specific characteristics of the DeskCycle that influence feasibility as well as some potential areas for improvement. Limitations of the study included lack of generalizability of the results. Our inclusion criteria and setting resulted in a predominantly female sample, which led to including only women in the study. Missing data from non-response and dropouts was a challenge as the dropouts might have had different experiences with the DeskCycle and its feasibility. Reasons for dropping out of the study were sought, and only two were linked to the DeskCycle i.e., it did not fit in the participant’s workstation. All other reasons were personal or work-related changes.

## Conclusion

Overall, the DeskCycle is a feasible intervention to reduce SB in the office environment. The DeskCycle positively impacted many domains of participants’ lives, including increased physical activity and positive feelings. Future studies should include a behavioral component to the intervention to evaluate its impact on adherence to recommended DeskCycle use and potential to be part of sustainable large-scale employee wellness programs.

## Supporting information

S1 File (XLSX)
